# A comparison of the histopathologic pattern of the left atrium in canine dilated cardiomyopathy and chronic mitral valve disease

**DOI:** 10.1186/s12917-015-0626-z

**Published:** 2016-01-05

**Authors:** Izabela Janus, Agnieszka Noszczyk-Nowak, Marcin Nowak, Rafał Ciaputa, Małgorzata Kandefer-Gola, Urszula Pasławska

**Affiliations:** Division of Pathomorphology and Veterinary Forensics, Department of Pathology, Wroclaw University of Environmental and Life Sciences, Wroclaw, 50375 Poland; Department of Internal Medicine and Clinic of Diseases of Horses, Dogs and Cats Wroclaw University of Environmental and Life Sciences, Wroclaw, 50366 Poland

**Keywords:** Dilated cardiomyopathy, Mitral valve disease, Dog, Left atrium, Histopathology

## Abstract

**Background:**

Dilated cardiomyopathy (DCM) and chronic mitral valve disease (CMVD) in dogs are associated with heart chamber enlargement, also of the left atrium. DCM is often accompanied by rhythm disturbances (mainly atrial fibrillation or ventricular arrhythmias). In CMVD, arrhythmias are observed less frequently. It is still unclear whether left atrial enlargement in these diseases results from volume overload or if it is also connected with other factors (e.g. rhythm disturbances).

This study was conducted on the left atrial myocardial specimens from 31 dogs, including those from 16 dogs with clinically diagnosed DCM and 15 dogs with CMVD. After fixation and staining (using haematoxylin-eosin and Masson-Goldner trichrome stain), the specimens underwent evaluation. Parenchymal changes (fibrosis, fatty infiltration, and vessel narrowing), degenerative changes (loss of striation, changes in cardiomyocyte structure, and abnormal cell nuclei) and the presence of inflammatory infiltrates were assessed.

**Results:**

More interstitial fibrosis (median 4 vs. 2.5 grid fields; *p* < 0.05) and less perivascular fibrosis (median score 1 vs. 2; *p* < 0.05) was observed in the DCM group compared to the CMVD group. Moreover, less distinct vessel narrowing was observed in the DCM group than in the CMVD group (median lumen area ratio 0.3 vs. 0.26 respectively; *p* < 0.05). Dogs with DCM showed more strongly defined degenerative changes than the CMVD dogs (median nuclei enlargement score 3 vs. 1, median loss of striation score 3 vs. 2 and median structural alterations score 3 vs. 2, respectively; *p* < 0.05).

**Conclusion:**

The obtained results indicate a different nature of changes occurring in the left atrial myocardium of dogs with DCM compared to dogs with mitral valve disease, including differences in vessel narrowing, cardiomyocyte degeneration and in the distribution of connective tissue.

## Background

Dilated cardiomyopathy (DCM) is a polyetiological disease characterised by a dilation of heart chambers (mainly the left ventricle and left atrium) without other significant structural heart defects. In some cases, it is combined with myocardial failure [1, 2]. It is mostly encountered in large and giant breed dogs but it can also develop in dogs of other breeds (e.g. English and American cocker spaniels, poodles and Portuguese water dogs) [1–7]. During the development of DCM the radiographic, echocardiographic and post-mortem examinations reveal an enlargement of the left ventricle and left atrium or an enlargement of all the heart chambers [1, 2, 4, 5, 7]. The ventricular dilation in DCM alters the geometry of atrioventricular valves, which results in valvular insufficiency. Mitral regurgitation increases left atrial pressure that may worsen the pathological remodelling of the left atrium and the left ventricle and independently stimulate the dilation of the heart chambers [8]. The disease is often accompanied by rhythm disturbances, mainly atrial fibrillation. However, there is no consent whether atrial fibrillation is the cause or result of atrial enlargement [9].

The histopathologic examination of the samples obtained from dogs with DCM demonstrates changes in the myocardial structure. There are two well-described forms of histopathologic lesions in the ventricular specimens of dogs with DCM: the attenuated wavy fibre type (AWF) and the fatty infiltration-degenerative (FID) type. Those lesions are mostly encountered in specimens from the left ventricle, but can also occur in the myocardium of the right ventricle and interventricular septum [1, 2, 4, 5, 8, 10]. Our previous study showed similar changes in atrial specimens from dogs with FID types of lesions in the ventricles. In dogs with the AWF type of ventricular lesions, the changes observed in atria can resemble either AWF or FID types of changes [11]. The attenuation of cardiomyocytes is a specific pathological feature of DCM. However, vacuolisation, extensive fibrosis and fatty infiltration may also occur in the ventricular walls during the course of that disease [1, 2, 4, 7]. Due to the fact that there is a similarity between the FID type of DCM and arrhythmogenic right ventricular cardiomyopathy, it is important to accurately characterise the disease based on the clinical and histopathological examinations [5].

The mechanism of changes occurring in the atrial myocardial structure in DCM is not well defined. It is suspected that the structural protein defects, impaired tissue oxygen supply and other factors may play a role in the development of the disease [1, 2]. It is not well understood whether the macroscopic and microscopic changes within the atria in dogs with DCM are a result of primary remodelling, the presence of arrhythmias, or a response of the cardiac tissue to functional disturbances, including volume overload (which also occurs in mitral valve disease).

Canine chronic mitral valve disease (CMVD) is the most common cardiovascular disease in dogs and the most frequent cause of congestive heart failure in those animals. The disease predominantly occurs in small breed dogs (e.g. Cavalier King Charles spaniels, Yorkshire terriers, dachshunds) and progresses with age [12–14]. The progressive dilation of the left ventricle and the left atrium in CMVD results from degenerative changes and an insufficiency of the mitral valve, leading to symptoms of heart failure [15]. Histopathologic changes within the ventricular walls include fibrosis and arteriosclerosis without or with slight myocyte atrophy [16, 17].

The purpose of the study was a histopathologic comparison of the left atrial wall specimens obtained from dogs with severe left atrial enlargement resulting from clinically diagnosed dilated cardiomyopathy (DCM) or chronic mitral valve disease (CMVD). To the authors’ knowledge, there are no detailed studies comparing histopathologic changes occurring in the atria in those diseases.

## Methods

### Case selection

Thirty-one dogs aged from 4 to 19 years (23 males and 8 females) with either CMVD or DCM were included in the study. Dogs met the inclusion criteria if they had an enlarged left atrium according to an echocardiographic examination (left atrial (LA)-to-aortic root (Ao) diameter ratio; LA/Ao >1.7 [18]), which was confirmed in a post-mortem examination. Dogs were included in the DCM group if they had a dilated left ventricle and a decreased fractional shortening (FS < 20 %) noted during an echocardiographic examination [1, 2, 5] with normal or type 1 mitral valve lesions (according to Whitney [19]). Dogs were included in the CMVD group if they had preserved left ventricular systolic function (FS > 20 %, noted in the echocardiographic examination) with type 3 or 4 mitral valve lesions (according to Whitney [19]). At the time of death or euthanasia, all dogs presented with stage D heart failure (according to the ACVIM Consensus Statement [20]).

### Ante-mortem examination

All animals underwent a clinical examination, echocardiography and electrocardiography, and were treated and re-examined every 6 months, until they died or were euthanized due to heart failure refractory to treatment. Only the LA/Ao ratio from the last examination of each dog served as an inclusion criterion.

The ECG was performed in right lateral recumbency using a 6-channel BTL SD08 device (BTL, UK) with I, II, III, aVL, aVR, aVF, and precordial (V1-V6) lead recordings. The echocardiographic examination was performed in standard views using the Aloka SSD 4000 and Aloka Alpha7 machines and included the assessment of the LA/Ao ratio, end-systolic and end-diastolic left ventricular measurements, ejection fractions and FS, blood velocity through the aortic and pulmonic valve, and the function of the atrioventricular valves. The relative left atrial size was estimated based on the LA/Ao. This parameter was determined using standard images acquired from a right short-axis view at the base of the heart. Estimates of left ventricular systolic function were obtained from the index of circumferential myocardial contraction and FS using the formula: FS[%] = ([LVIDd – LVIDs]/LVIDd) ×100, where LVIDd and LVIDs are the internal left ventricular dimensions at end-diastole and end-systole, respectively. Left ventricular dilation was estimated based on the comparison of chamber measurements (internal diameter and wall thickness) to standard breed-specific or body-weight specific values [21]. The function of the atrioventricular, pulmonary and aortic valves was examined using pulsed wave Doppler and color Doppler techniques.

### Post-mortem examination

The results of the echocardiographic examination (left atrial and left ventricular enlargement) were confirmed during the post-mortem examination, where the type of mitral valve lesion was determined. Heart measurements were taken using a manual 150 mm Beerendonk caliper, measured to the nearest 1/20 mm. The measurements were taken in planes compatible with those used during the echocardiographic examination. Specimens from the left atrial wall were collected from each dog for further histopathologic analysis. They were fixed in 7 % buffered formalin, embedded in paraffin blocks, sectioned at 6 μm, stained using the standard haematoxylin-eosin (H&E) method and Masson-Goldner trichrome (for a better visualisation of connective tissue), and underwent a light microscopy evaluation. Photomicrographs of each studied specimen were subjected to computer-assisted image analysis, using a computer coupled to an optical Olympus BX53 microscope, equipped with a Color View IIIu digital Olympus camera (Olympus, Japan). A morphometric analysis was performed using Cell^A software (Olympus Soft Imaging Solution GmbH, Germany).

To accurately describe the histopathological changes in the atria, specimens were evaluated for the presence and severity of fibrosis, fatty infiltration, inflammatory infiltration, intra-myocardial arterial narrowing and degenerative changes in cardiomyocytes. The latter was expressed as the presence of abnormal cell nuclei (swollen nuclei with an altered structure surrounded by a “halo”), loss of striation, and other features of degeneration (e.g. swelling of the cardiomyocyte, blurring of the cell structure and alterations in staining).

The presence and severity of interstitial fibrosis and fatty infiltration were evaluated after superimposing a 16-field grid over the digital image and counting the number of fields that showed the presence of fatty infiltration or fibrosis (in 200× magnification, after Masson-Goldner trichrome staining), as proposed by Lobo et al. [7].

The severity of perivascular fibrosis was assessed using the following scoring system: 0, no fibrosis; 1, mild; 2, moderate; 3, severe fibrosis (in 100× magnification, after Mason-Goldner trichrome staining).

Inflammatory infiltrates were evaluated according to the number of inflammatory cells in a field at a 400× magnification (H&E staining).

Intra-myocardial arterial narrowing was assessed using the lumen area ratio (LAR; the luminal area of the vessel divided by the total vessel area, not including the adventitia), as proposed by Falk et al. [16] (at 200× magnification, after H&E staining).

Cardiomyocyte degenerative changes (abnormal cell nuclei, loss of striation and changes in cardiomyocyte structure) were assessed using the following scoring system: 0, none or involving <25 % of the myofibres; 1, involving 25–50 % of the myofibres; 2, involving 50–75 % of the myofibres; 3, involving >75 % of the myofibres (at 400× magnification, after H&E staining).

Each of the features was evaluated in 20 randomly chosen fields per slide (for fatty infiltrates, interstitial fibrosis, inflammatory infiltrates, and cardiomyocyte degenerative changes) or in at least 10 vessels per slide (for perivascular fibrosis and LAR), and a mean score was subsequently calculated for the slide.

### Ethical consideration

According to the Polish law [22], standard diagnostic procedures and studies conducted on animal tissue do not require permission from the Ethical Board. Oral consent for the ante-mortem examination (electrocardiography and echocardiography) was obtained from all owners, as those examinations are used routinely to diagnose canine heart disease.

### Statistical analysis

A statistical analysis was performed using StatisticaPL® software (StatSoft, Poland). The Shapiro-Wilk analysis was used to test the data normality. The Mann–Whitney *U* test (for data distributed non-normally) or Student’s t test (for data distributed normally) were used to compare groups. The level of statistical significance was set at *P* < 0.05.

The results are shown as range and median values.

## Results

The DCM group consisted of 16 dogs (9 Doberman pinschers, 4 German shepherds, 2 boxers, and 1 great Dane, aged 4–11 years, including 12 males and 4 females). Seven specimens from the left ventricle presented with an AWF type of lesion, while nine specimens had features of a FID type of lesion. The CMVD group comprised 15 dogs (10 mixed-breed dogs, 2 dachshunds, 1 Cairn terrier, 1 miniature pinscher, and 1 German pinscher, aged 8–19 years, including 11 males and 4 females) diagnosed with mitral valve disease combined with an enlarged left ventricle and left atrium (Table 1).

**Table 1:** Population study and clinical findings in the examined groups

	DCM group	CMVD group	*p*-value
	*n* = 16	*n* = 15	
age (median; range) [years]	9 (4–11)	15 (8–19)	<0.001
weight (median; range) [kg]	31 (30–45)	11 (5–25)	<0.001
arrhythmia (*n*)	16	4^a^	
atrial fibrillation (*n*)	15	1	
ventricular tachycardia (*n*)	1	-	
atrial premature complexes (*n*)	-	2	
ventricular premature complexes (*n*)	-	2	
LA/Ao (mean ± SD)	3.14 ± 0.31	2.44 ± 0.71	0.08

*LA/Ao* left-atrial-to-aorta ratio, *SD* standard deviation

^a^one dog showing both single atrial premature complexes and ventricular premature complexes

Dogs in the DCM group were significantly younger and heavier than those from the CMVD group, but there was no difference in the LA/Ao ratio between both groups (Table 1). All dogs in the DCM group and 26.7 % of dogs in the CMVD group had rhythm disturbances, as shown in Table 1.

**Fig. 1:**
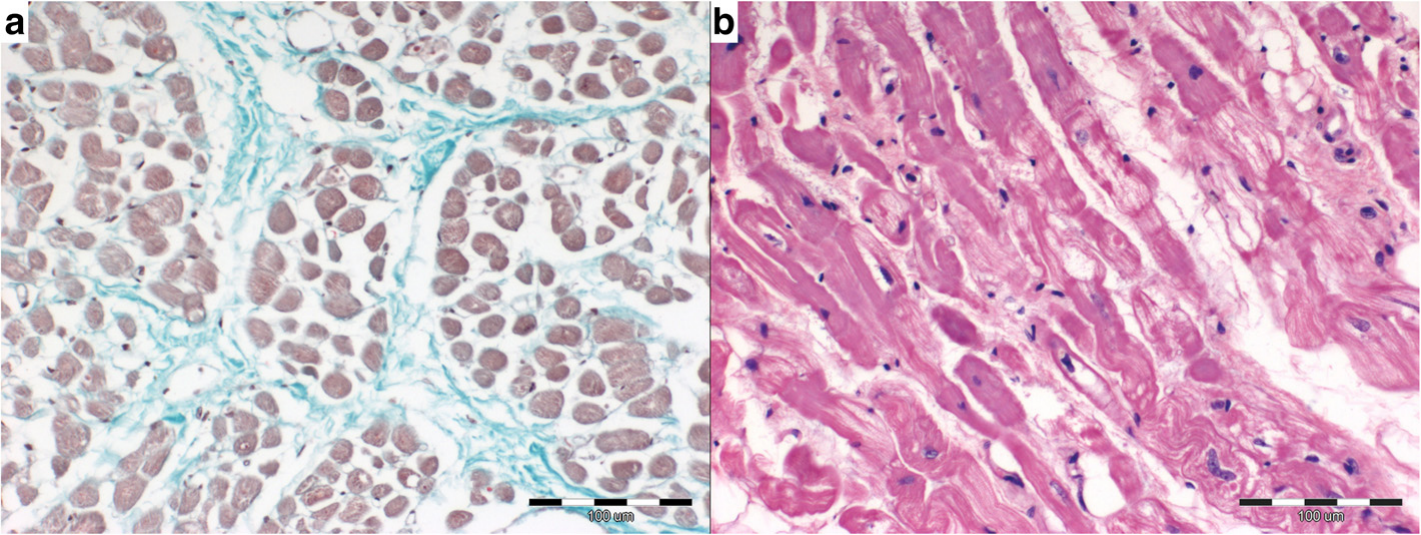
Histopathologic pattern of atrial myocardium in DCM group. **a** interstitial fibrosis (Masson-Goldner trichrome), **b** myocardial degeneration presented by changes in cardiomyocyte structure, loss of striation and changes in cell staining (H&E)

**Fig. 2:**
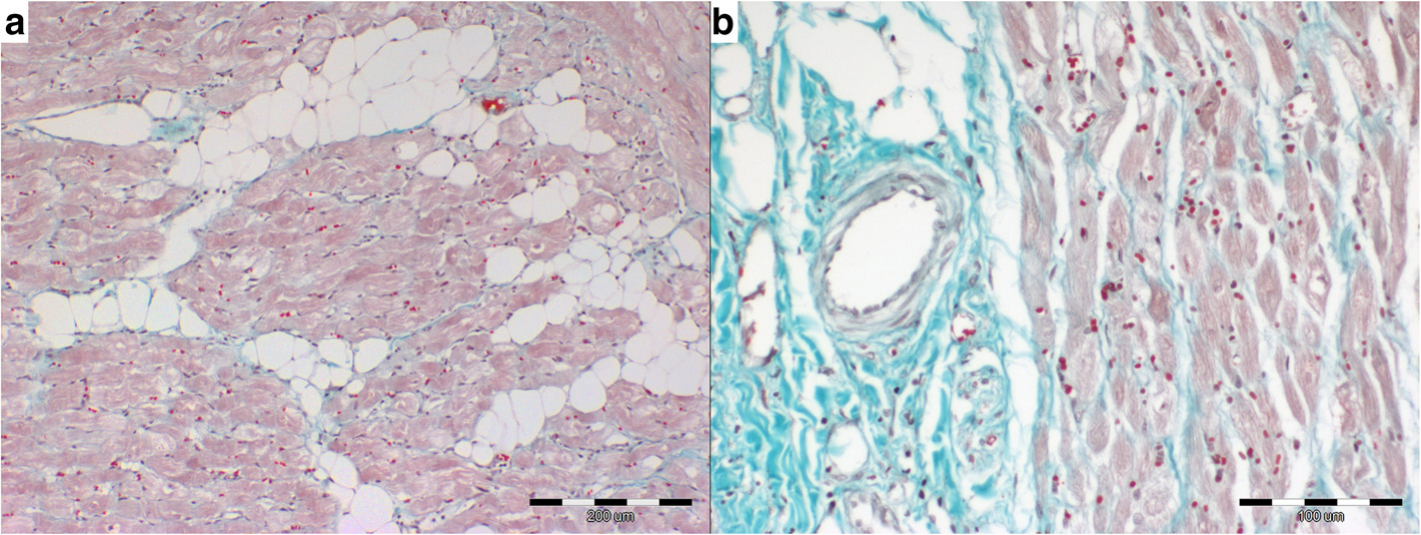
Histopathologic pattern of atrial myocardium in CMVD group. **a** fatty infiltration with slight amount of interstitial fibrosis (Masson-Goldner trichrome), **b** perivascular fibrosis with slight amount of interstitial fibrosis (Masson-Goldner trichrome)

The results of the histopathological analysis are shown in Table 2. The DCM group showed significantly more interstitial fibrosis, less perivascular fibrosis, a higher LAR value, and no difference in fatty infiltration severity compared to the CMVD group (Figs. 1 and 2). Moreover, we noted a significantly more cardiomyocyte degenerative changes in the DCM group compared to the CMVD group (Figs. 1 and 2). They included the presence of abnormal cell nuclei, loss of striation and changes in the structure of the cardiomyocytes. No difference in the severity of inflammatory infiltrates was noted between both groups. The inflammatory infiltration consisted of single lymphocytes mainly localised subendocardially. The average number of inflammatory cells per field did not exceed 2 in any of the examined specimens.

**Table 2:** Histopathological findings in the examined groups

	DCM group	CMVD group	*p*-value
	*n =* 16	*n* = 15	
interstitial fibrosis (median; range)	4 (1–7)	2.5 (1–5)	0.036
perivascular fibrosis (median; range)	1 (0–2)	2 (1–2)	0.006
fatty infiltration (median; range)	1 (0–10)	1 (0–6)	0.663
LAR (median; range)	0.3 (0.14–0.8)	0.26 (0.15–0.33)	0.041
abnormal cell nuclei (median; range)	3 (1–3)	1 (0–3)	0.008
loss of striation (median; range)	3 (1–3)	2 (0–3)	0.004
altered cardiomyocyte structure (median; range)	3 (2–3)	2 (0–3)	0.004
inflammatory infiltration (median; range)	0.36 (0–1.6)	0.35 (0–1.9)	0.796

*LAR* lumen area ratio

## Discussion

There have been numerous histopathological characterisations of canine DCM [1, 2, 4, 7]. However, changes occurring in the atrial tissue in the course of DCM remain poorly described. Similarly to our findings, Everett et al. [8] noted fibrosis and cardiomyocyte atrophy in specimens from the atria in Doberman pinschers with DCM. Those lesions were less extensive and less common than the lesions found in specimens from ventricles in the same animals.

According to Schaper et al. [23], normal cardiac tissue in failing hearts in humans is replaced by fibrosis. This is accompanied by changes within the cardiomyocyte nuclei (including nuclear enlargement). Sharov et al. [24] noted a disruption in the structure of cardiomyocytes with subsequent fibrosis in dogs with heart failure. The authors associated myocardial fibrosis with changes in the expression of structural proteins. We observed lesions in cardiomyocytes and interstitial tissue in both groups, which may reflect the response of the heart to volume overload, as described in the abovementioned studies. In our study, there was more interstitial fibrosis and nuclear enlargement in the DCM group.

When comparing ventricular specimens from dogs showing DCM and CMVD, Falk et al. [17] noticed that the majority of dogs with CMVD showed only a small degree of cardiomyocyte atrophy in the ventricular specimens or none at all. Such differences in the severity of cardiomyocyte degeneration were also observed in our study.

Studies on dogs with CMVD showed that animals developing congestive heart failure present with lower values of LAR as compared to controls, although this parameter is highly variable in each dog [16]. Falk et al. [17] showed that a decrease in LAR is directly associated with a decrease in fractional shortening in the left ventricle. This suggests the presence of alterations in intra-myocardial vessels in the course of heart dilation in CMVD. Although LAR is not a highly reliable parameter [17], its greater values observed in the DCM group in our study together with lower values of perivascular fibrosis may indicate that alterations in intra-mural vessels play a greater role in left atrial enlargement in CMVD than in DCM.

DCM in dogs is often connected with the occurrence of supraventricular and ventricular rhythm disturbances. Sustained arrhythmias in CMVD occur less frequently [1, 2, 5, 25, 26]. Noszczyk-Nowak et al. [27] and Crosara et al. [26] demonstrated a higher risk of developing supraventricular arrhythmias in dogs with an enlarged left atrium, although this phenomenon is not well understood. It has not been established whether arrhythmias, also found in dogs in pre-clinical stages of DCM, are a consequence of primary myocardial changes or if myocardial lesions result from long-lasting rhythm disturbances and secondary heart chamber dilation [5, 28].

Recent studies [28–30] emphasize the role of myocardial fibrosis in the development and progression of atrial fibrillation (AF) of different aetiology in humans. Platanov et al. [30] showed a significantly higher amount of connective tissue in heart specimens from patients with AF, compared to patients without atrial rhythm disturbances. Nattel and Harada [31] highlighted the possible role of fibrosis in cardiac conduction disturbances.

Frustaci et al. [32] noted changes in the atrial myocardial structure (inflammation, non-inflammatory cardiomyopathy or fibrosis) in patients with lone atrial fibrillation without coexisting heart chamber dilation (including atrial diameter). Moreover, Tuomainen et al. [33] noted significantly lower values of the left ventricular end-diastolic diameter, left ventricular end-diastolic volume and left ventricular end-systolic volume combined with a significantly higher value of the left atrial diameter in patients with chronic AF in DCM compared to patients with DCM and a sinus rhythm. Considering that all dogs with DCM in this study had an arrhythmia, it is impossible to determine whether the observed changes are a consequence of the primary myocardial disease or a consequence of the arrhythmia.

One of the limitations of our study was a lack of dogs with DCM without rhythm disturbances, which could be compared to dogs included in the study. Dogs with DCM often develop rhythm disturbances before the development of clinical symptoms. Further studies including dogs with DCM without arrhythmias may improve our knowledge of the pathogenesis of this disease.

Another limitation was an observed difference between body weight, age and breed distribution in both groups. This was a result of the different clinical courses of both diseases. DCM mostly occurs in young large-breed dogs, while CMVD mostly occurs in old small-breed dogs. To minimize those differences, we used an increased LA/Ao ratio as an inclusion criterion. There was no difference in the LA/Ao ratio between both groups. Therefore, we assumed that the comparison of atrial remodelling in both groups was justifiable.

The variety in the DCM group was a further limitation of the study. We included dogs with idiopathic DCM that developed arrhythmias in the course of the disease and dogs presenting with AF in the first examination. The latter dogs may have had tachycardia-induced cardiomyopathy. However, all the dogs in the DCM group did not respond well to treatment in late stages of the disease. They developed severe heart failure with left atrial enlargement, and presented with severe tachyarrhythmia at the time of death or prior to euthanasia. Hence, they were included in one group.

The last limitation was a lack of assessment of atrial pressure in the examined dogs prior to the histopathologic examination in order to exclude its influence on atrial remodelling. We attempted to minimise the possibility of other reasons for atrial enlargement (e.g. mitral stenosis) by performing an accurate ante-mortem and post-mortem examination.

## Conclusion

Dogs with dilated cardiomyopathy show a different distribution of connective tissue, have less severe intra-myocardial arterial narrowing, and have more severe degenerative changes in the cardiomyocytes of the left atrium compared to dogs with chronic mitral valve disease. The changes noted in the atrial tissue from dogs in both groups resemble lesions noted in the ventricular tissue of dogs with the same diseases. Those differences may indicate that the atrial enlargement noted in DCM and CMVD has a different, disease-specific underlying mechanism and does not result only from volume overload.

## Abbreviations

AF: atrial fibrillation
AWF: attenuated wavy fibres type
CMVD: chronic mitral valve disease
DCM: dilated cardiomyopathy
FID: fatty infiltration-degenerative type
FS: fractional shortening
H&E: haematoxylin-eosin stain
LA/Ao: left atrial-to-aortic root diameter ratio
LAR: lumen area ratio
LVIDd: end-diastolic left ventricular internal diameter
LVIDs: end-systolic left ventricular internal diameter
